# Toward an open access genomics database of South Africans: Legal considerations

**DOI:** 10.17159/sajs.2023/15069

**Published:** 2023-08-08

**Authors:** Donrich Thaldar, Amy Gooden, Dusty-Lee Donnelly

**Affiliations:** 1 School of Law, University of KwaZulu-Natal, Durban, South Africa; 2 Petrie-Flom Center for Health Law Policy, Biotechnology, and Bioethics, Harvard Law School, Cambridge, Massachusetts, USA

**Keywords:** data protection, genomics, open science, Research Code of Conduct

## Introduction

Why should individual-level genomic data be private? Concerns range from unintentionally discovering previously unknown family members to insurance discrimination based on health risks disclosed by the genomic data. Although many research participants express concern about the privacy of their genomic data, the picture of the nature and extent of their concerns is a complex and highly variable one.^1,2^ Also, there are persons who are willing to share their genomic data in the public domain without requiring any privacy guarantees.^1–5^ An iconic example is the Harvard Personal Genome Project (Harvard PGP) initiated in 2005^6^, which has since become a global network of projects.^7^ It publishes the whole genome sequences of its research participants online for anyone around the world to download^6^ – no registration required, no paywall, and no data access committee. This is a truly open access, individual-level genomic database. Furthermore, its research participants may choose to supplement their genome sequences by also including phenotype and health information in the open access database.^6^

Imagine a South African version of the Harvard PGP, i.e. an open access, individual-level genomic database composed of the genomic data of thousands of South Africans, freely available to all. Advances in science and technology have resulted in improvements in the time, cost, and methods involved in genome sequencing^8,9^, and the focus has now shifted to filling the gaps in the amount, and reliability, of population-level data about newly discovered genes and their links to disease. However, the success and effectiveness of various medicines and therapies, as well as the realisation of precision medicine, may be hampered by differences in the reference group and population on which clinical trials are conducted. This poses a real concern for countries like South Africa, whose major population groups are grossly underrepresented in existing genomic reference sets.^10–14^ Establishing an *inclusive, open access genomics project* would not only align with all the benefits typically associated with open science, but may also offer a solution to the problem of underrepresentation.

But, is there a legal pathway to establishing such an *open access* genomics project in South Africa? In this article, we explore this question from the perspective of the *Protection of Personal Information Act 4 of 2013* (POPIA). Furthermore, the Academy of Science of South Africa (ASSAf) has recently submitted its long-awaited proposed Code of Conduct for Research (proposed CCR)^15^, in terms of POPIA, to the Information Regulator. As such, where relevant, we make recommendations on how the proposed CCR should be amended to clarify the relevant law and provide guidance with regard to such an open access genomics project(s).

## Terminology

A core concept in this article is *research*. As POPIA does not define research, it should be understood in its general meaning. A widely used definition of research is that of the Organisation for Economic Cooperation and Development (OECD), which reads as follows: ‘Any creative systematic activity undertaken in order to increase the stock of knowledge, including knowledge of man, culture and society, and the use of this knowledge to devise new applications.’^16^ The proposed CCR (at paragraph 1.1.2.3.1.) offers a definition of research that is very similar to the OECD definition, namely that research ‘includes the range of activities that a private or Public Body conduct to extend knowledge through disciplined enquiry or systematic investigation’^15^. The only problem with the proposed CCR’s definition is that it seems to exclude individual researchers who are not part of a ‘private or public body’, while the rest of the proposed CCR clearly contemplates the inclusion of such individual researchers. We suggest that the proposed CCR should be revised to include independent individual researchers within the ambit of its definition of research. This can be accomplished either by explicit inclusion of independent individual researchers in the definition, or by removing the reference to the entities that conduct research – in line with the OECD definition.

## Analysis

### Are individual-level genomic data regulated by POPIA?

The first part of the POPIA analysis is to establish whether individual-level genomic data fall under the two main types of information regulated by POPIA, namely personal information and special personal information:

- Personal information is information that relates to, inter alia, an ‘identifiable, living, natural person’ (section 1 of POPIA). As genomic data relate to a living natural person, and the person can be identified using the genomic data, genomic data qualify as *personal information*.
- Special personal information is a subclass of personal information that relates to, inter alia, a natural person’s race, health, or biometric information (section 26 of POPIA). Genomic data relate to all three of these and therefore clearly qualify as *special personal information*.

Accordingly, individual-level genomic data are indeed regulated by POPIA at two levels: frst at the level of personal information, and second at the level of special personal information.^17^

Importantly, regulation of individual-level genomic data by POPIA commences from the moment that the genomic data are *generated* through sequencing and recorded in an electronic device (section 3(1)).^17,18^ POPIA does not apply to biological samples or to DNA.^17,18^ This is because POPIA applies only to personal information that is *entered in a record*, while genomic information is naturally present in DNA, rather than being entered in DNA.^17,18^ Individual-level genomic data, once generated and recorded, will likely always fall within POPIA, as it seems unlikely that such genomic data can be de-identified.^19,20^

### Uploading genomic data to an open access database

After individual-level genomic data are generated, the next step in the context of an open access genomics project would be for the project to upload data subjects’ genomic data to the project’s open access online database. This would qualify as *processing* the genomic data in terms of section 1 of POPIA (which includes ‘any operation or activity or any set of operations’ such as collecting, recording, organising, storing, disseminating, or making available in any other form). As a general rule, the processing of personal information and special personal information is only lawful if a legal ground for processing is present. *Consent* is a legal ground for processing in the case of both personal information (section 11(1)(*a*) of POPIA) and special personal information (section 27(1)(*a*) of POPIA). Consent is defined by POPIA as a ‘voluntary, specific, and informed expression of will’ (section 1). Accordingly, to be POPIA compliant, data subjects must voluntarily agree to the uploading of their genomic data to the open access online database, while understanding the consequence – that this will make their genomic data public – and the possible privacy risks thereof – that their genomic data contains information of a personal nature from which they can be identified.^21–24^ For consent to be informed, data subjects would also need to understand that this will impact upon their data subject rights (section 5 of POPIA), including their rights in relation to the cross-border transfer of their data (sections 57 and 72 of POPIA), discussed below. But how does one know whether data subjects understand this, and whether their consent is therefore truly *informed*?

The Harvard PGP’s solution was to develop a new consent model that they called *open consent*.^25,26^ This entails, inter alia, that prospective participants are provided with resource material that explains not only the benefits to science of participating in the Harvard PGP, but also the potential risks to participants of being identified through their open genomic data.^27^ In contrast with most research projects that do not assess whether prospective participants objectively understand what they are consenting to, open consent requires prospective participants to take an online entrance examination to *objectively assess their understanding*.^26,27^ This examination can be taken repeatedly, but only prospective participants who achieve full marks are admitted as participants in the Harvard PGP.^26,27^ In other words, the rationale behind open consent is that the heightened risk to privacy (by making one’s genomic data open access) is offset by the heightened measure of objective assessment to ensure that consent is truly *informed*.

We suggest that objective assessment of understanding should be a requirement for open access genomics projects in South Africa, given the heightened risk to data subjects. Provided that this is complied with, i.e. that data subjects voluntarily agree to the uploading of their genomic data to the open access online database and pass an objective assessment showing that such consent is informed, the processing will be lawful.

### Accessing the data subject’s genomic data on the Internet

Once the genomic data are published on the Internet, anyone can access and use the data for research or for any other purpose, as the data are open access. This would qualify as further processing of the genomic data in terms of section 15 of POPIA. Again, as a general rule, (further) processing of personal information and special personal information is only lawful if a legal ground for such processing is present. One such legal ground for (further) processing of both personal information (section 15(3)(*b*) of POPIA) and special personal information (section 27(1)(*e*) of POPIA) is if information has *deliberately been made public by the data subject*.

This raises the question: must data subjects themselves perform every action necessary to make the relevant information public, or can other persons act as their agents? Although not covered in POPIA, the proposed CCR provides for situations where data subjects ‘consented’ that an ‘intermediary’ can intentionally make their personal information public.^15^ This is a welcome provision in the proposed CCR. However, from a legal perspective, it would be better to use the stronger word ‘instruct’, as this would imply the nominate contract of ‘mandate*’*, which entails that one party (the mandatee) gratuitously performs a service for the other party (the mandator).^27–30^ This nominate contract originates from Roman law, and automatically entails that the mandatee must exercise reasonable care when performing the mandate on instruction of the mandator.^29,30^ We suggest that a mandate construction is essential to comply with sections 15(3)(*b*) and 27(1)(*e*) of POPIA.

Accordingly, it would be important in the context of open access genomics projects that data subjects not only *consent* to the uploading of their genomic data to an open access online database, and hence to making it public, but simultaneously also *instruct* the open access genomics project to perform said action. If this is done, the open access genomics project has a mandate to make the data subjects’ genomic data public. Given that mandate is such a well-established part of South African law, this should suffice for compliance with sections 15(3)(*b*)) and 27(1)(*e*) of POPIA respectively. As a consequence, anyone would be able to lawfully use the genomic data, thus succeeding in the open science objective of the open access genomics project.

### The cross-border aspect

An open access genomics project would make data openly accessible on the Internet – which means that the data would be available beyond physical geographical borders, and may thus bring about the provisions regarding transfers of personal information outside of South Africa in section 72 of POPIA. If the genomic data are stored on a server outside of South Africa, as is the case with many cloud services, this in itself would constitute a cross-border data flow. Further, whenever the data are downloaded outside of South Africa, there is a cross-border data flow. POPIA’s provisions on cross-border data flows thus apply.

The cross-border transfer of personal information may only take place if, inter alia, the transfer is required in terms of a *contract* between the data subject and the responsible party (section 72(1)(*c*) of POPIA). As we have suggested above, data subjects should *instruct* the open access genomics project to upload their genomic data to its open access online database, as this would constitute the *contract* of mandate. Accordingly, the project giving access to anyone anywhere in the world to download the data would be in pursuance of the terms of the *contract*, and hence comply with POPIA’s regime for the cross-border transfer of personal information.

However, because genomic data are not only personal information, but also special personal information, there is an additional requirement, namely that if a party who downloads the data are in a country that is not deemed to provide adequate protection for the processing of personal information, the open access genomics project must obtain prior authorisation from the Information Regulator (section 57(1)(*d*) of POPIA). Given that the South African Information Regulator has not yet issued a list of countries that it deems as providing adequate protection, no country currently qualifies as such. Also, even if the Information Regulator issues such an adequacy list, the purpose of the open access genomics project is to make its genetic data easily available to anyone in the world, regardless of whether the recipient is in a country that is deemed to provide adequate protection or not. Accordingly, the open access genomics project would need to apply for prior authorisation from the Information Regulator in order to comply with POPIA.

Importantly, if a code of conduct has come into force in the relevant sector of society, the prior authorisation requirement for the cross-border transfer of special personal information ceases to apply (section 57(3) of POPIA). Accordingly, if and when the proposed CCR comes into force, there will be respite for open access genomics projects in this regard. This highlights the importance of having sufficient provisions in the proposed CCR to properly regulate open access genomics projects.

### Various rights of data subjects

Data subjects giving consent and instructing the open access genomics project to upload their genomic data to an open access online database do not exhaust the data subjects’ rights from the perspective of POPIA. We briefly analyse other relevant rights of data subjects in the context of open access genomics projects and consider how these rights apply in the context of an open access genomics project.

First, data subjects would have the right to request (in terms of section 23 of POPIA) information from the open access genomics project about the identity of all third parties who have access to their genomic data. We suggest that this would place a duty on the open access genomics project: (1) to require would-be data downloaders to first register on the project website; and (2) to take reasonable measures – such as a verification email – to verify the registration information. Should data subjects exercise their right to request information about the identity of all third parties who have access to their genomic data, the open access genomics project would be in a position to provide this to them.

Second, data subjects would have the right to be notified of, inter alia, the data being collected, the identity of the responsible party, and the purpose of collection (section 18(1) of POPIA). An exception to this right is when data are collected for the purpose of research (section 18(4)(*f*) (*ii*) of POPIA). Also, data subjects can waive this right if they consent to non-compliance with the notification requirement (as provided for in terms of section 18(4)(*a*) of POPIA). Accordingly, an open access genomics project would have two options: (1) expand the registration requirement mentioned above by requiring would-be data downloaders to declare that they intend to use the data for research; and (2) incorporate a waiver of the notification right in the consent process for data subjects. Given that (1) does not provide any guarantees, we suggest that the best solution would be to implement both (1) and (2).

Third, data subjects would have the right to withdraw their consent at any time (section 11(2)(*b*) of POPIA), to object to the processing of their data on reasonable grounds (section 11(3)(*a*) of POPIA), and to request that their data be deleted (section 24 of POPIA). In the context of an open access genomics project, this would require that the project removes the data from its website. However, the project would not be under any obligation to take steps to have the data deleted by others who have already downloaded such data. In this regard, the Harvard PGP promises to take an extra step, namely that they will ‘request any organizations or researchers with whom the PGP has any formal data sharing agreements to likewise delete your data and information within a reasonable time frame’^31^. We suggest that this would be a good policy to follow. Furthermore, the informed consent process should ensure that data subjects are aware of these rights, and how to exercise them.

## Conclusion and recommendations

Privacy is a right of persons to be exercised as they deem fit. Persons are autonomous moral agents, and provided that they understand the risks to their privacy, they should be free to make their own genomic data public. Moreover, as there is public benefit in such open sharing of genomic data, this is, in principle, something that should be welcomed from a public policy perspective. However, reasonable protective measures – aligned with POPIA – should still be put in place.

We recommend that the proposed CCR should clearly provide a roadmap for a prospective open access genomics project to follow. Flowing from our analysis in this article, we suggest the landmarks in this roadmap are the following:

1. *General*. The regulation of genomic data by POPIA commences from the moment that the genomic data are generated through sequencing and are recorded on an electronic device. POPIA does not apply to biological samples or to DNA.^18^ POPIA always applies to genomic data, as such data cannot be de-identified.
2. *Pertinent elements of consent*. The open access genomics project must provide resource material to prospective participants that explains, most pertinently in the context of POPIA: (1) what the open access genomics project entails and the possible risks to their privacy; and (2) their right to request information about persons who access their data, their right to withdraw, and the consequences of exercising these rights.
3. *Objective assessment*. The open access genomics project must require prospective participants to pass an objective assessment that assesses their understanding of the content of the resource material in order to ensure that their consent is truly *informed*.
4. *Consent plus mandate*. The open access genomics project must ensure that its participants: (1) consent to the uploading of their genomic data to an open access online database, and hence consent to making it public; and (2) instruct the open access genomics project to upload their genomic data to its open access online database.
5. *Registration*. The open access genomics project must: (1) require data downloaders to first register on the project website; (2) take reasonable measures, such as a verification email, to verify the registration information; and (3) require data downloaders to declare that they intend to use the data for research.

Note that the requirement for obtaining prior authorisation from the Information Regulator for cross-border transfers of data will fall away once a code of conduct for research is issued. As such, the suggested roadmap need not include prior authorisation. Implementing the above suggestions in the proposed CCR will provide clarity for the establishment of an open access genomics project, which in turn will benefit all South Africans.

## Significance:

There may be significant benefits to an open access genomics database of South Africans. The proposed Code of Conduct for Research should be amended to provide a clear roadmap – aligned with the *Protection of Personal Information Act 4 of 2013* – for open access genomics projects.

## References

[1] Clayton EW, Evans BJ, Hazel JW, Rothstein MA. The law of genetic privacy: Applications, implications, and limitations. J Law Biosci. 2019;6(1):1–36. 10.1093/jlb/lsz007 31666963 PMC6813935

[2] Wan Z, Hazel JW, Clayton EW, Vorobeychik Y, Kantarcioglu M, Malin BA. Sociotechnical safeguards for genomic data privacy. Nat Rev Genet. 2022;23:429–445. 10.1038/s41576-022-00455-y 35246669 PMC8896074

[3] Haeusermann T, Greshake B, Blasimme A, Irdam D, Richards M, Vayena E. Open sharing of genomic data: Who does it and why? PLoS ONE. 2017;12(5):1–15. 10.1371/journal.pone.0177158 PMC5423632 28486511

[4] Ball MP, Thakuria JV, Zaranek AW, Clegg T, Rosenbaum AM, Wu X, A public resource facilitating clinical use of genomes. Proc Natl Acad Sci USA. 2012;109(30):11920–11927. 10.1073/pnas.1201904109 22797899 PMC3409785

[5] Ball MP, Bobe JR, Chou MF, Clegg T, Estep PW, Lunshof JE, Harvard Personal Genome Project: Lessons from participatory public research. Genome Med. 2014;6(10):1–7. 10.1186/gm527 24713084 PMC3978420

[6] The Harvard Personal Genome Project. About [webpage on the Internet]. No date [cited 2022 Oct 15]. Available from: https://pgp.med.harvard.edu/about

[7] Personal Genome Project: Global network ‘The Personal Genome Project’ [homepage on the Internet]. No date [cited 2022 Oct 15]. Available from: https://www.personalgenomes.org/

[8] Adams J DNA sequencing technologies. Nat Educ. 2008;1(1):193. https://www.nature.com/scitable/topicpage/dna-sequencing-technologies-690/

[9] Preston J, VanZeeland A, Peiffer DA. Innovation at Illumina: The road to the $600 human genome [document on the Internet]. c2021 [cited 2022 Oct 15]. Available from: https://media.nature.com/original/magazine-assets/d42473-021-00030-9/d42473-021-00030-9.pdf

[10] Wits University. Why and how Africans need to participate in genetic studies [webpage on the Internet]. c2022 [cited 2022 Oct 27]. Available from: https://www.wits.ac.za/news/latest-news/research-news/2022/2022-02/why-and-how-africans-need-to-participate-in-genetic-studies-.html

[11] Adepoju P Tackling Africa’s underrepresentation in genomics studies. Nature Africa News. 2022 April 05. Available from: https://www.nature.com/articles/d44148-022-00051-6

[12] Jackson C Africa’s missing genomic data and its impact on health care. GEN. 2020 September 08. Available from: https://www.genengnews.com/insights/africas-missing-genomic-data-and-its-impact-on-health-care/

[13] Bentley AR, Callier SL, Rotimi CN. Evaluating the promise of inclusion of African ancestry populations in genomics. NPJ Genom Med. 2020;5:1–9. 10.1038/s41525-019-0111-x 32140257 PMC7042246

[14] Bentley AR, Callier S, Rotimi CN. Diversity and inclusion in genomic research: Why the uneven progress? J Community Genet. 2017;8(4):255–266. 10.1007/s12687-017-0316-6 28770442 PMC5614884

[15] Academy of Science of South Africa (ASSAf). Code of Conduct for Research [document on the Internet]. c2023 [cited 2023 Jun 06]. Available from: https://www.assaf.org.za/wp-content/uploads/2023/04/ASSAf-POPIA-Code-of-Conduct-for-Research.pdf

[16] Organisation for Economic Cooperation and Development (OECD). Frascati Manual: Guidelines for collecting and reporting data on research and experimental development [webpage on the Internet]. c2015 [cited 2022 Oct 15]. Available from: https://www.oecd.org/publications/frascati-manual-2015-9789264239012-en.htm

[17] Thaldar DW, Townsend BA. Exempting health research from the consent provisions of POPIA. Potchefstroom Electron Law J. 2021;24:1–32. 10.17159/1727-3781/2021/v24i0a10420

[18] Thaldar D Why POPIA does not apply to DNA. S Afr J Sci. 2021;117(7/8), Art. #11286. 10.17159/sajs.2021/11286

[19] Pike ER. Securing sequences: Ensuring adequate protections for genetic samples in the age of big data. Cardozo L Rev. 2015;37:1977–2033. https://ssrn.com/abstract=2658306

[20] Townsend BA, Thaldar DW. Navigating uncharted waters: Biobanks and informational privacy in South Africa. S Afr J Hum Rights. 2019;35(4):329–350. 10.1080/02587203.2020.1717366

[21] Gymrek M, McGuire AL, Golan D, Halperin E, Erlich Y. Identifying personal genomes by surname inference. Science. 2013;339(6117):321–324. 10.1126/science.1229566 23329047

[22] Erlich Y, Williams JB, Glazer D, Yocum K, Farahany N, Olson M, Redefining genomic privacy: Trust and empowerment. PLoS Biol. 2014;12(11):1–5. 10.1371/journal.pbio.1001983 PMC4219652 25369215

[23] Sweeney L, Abu A, Winn J. Identifying participants in the Personal Genome Project by name. SSRN. 2013:1–4. 10.2139/ssrn.2257732

[24] Malin B, Sweeney L. How (not) to protect genomic data privacy in a distributed network: Using trail re-identification to evaluate and design anonymity protection systems. J Biomed Inform. 2004;37(3):179–192. 10.1016/j.jbi.2004.04.005 15196482

[25] Lunshof JE, Chadwick R, Vorhaus DB, Church GM. From genetic privacy to open consent. Nat Rev Genet. 2008;9:406–411. 10.1038/nrg2360 18379574

[26] Zarate OA, Green Brody J, Brown P, Ramírez-Andreotta MD, Perovich L, Matz J. Balancing benefits and risks of immortal data: Participants’ views of open consent in the Personal Genome Project. Hastings Center Rep. 2016;46(1):36–45. 10.1002/hast.523 PMC4871108 26678513

[27] Angrist M Eyes wide open: The Personal Genome Project, citizen science and veracity in informed consent. Per Med. 2009;6(6):1–13. 10.2217/pme.09.48 22328898 PMC3275804

[28] Hutchison D, Pretorius CJ, Du Plessis J, Eiselen S, Floyd T, Hawthorne L, The law of contract in South Africa. 2nd ed. Cape Town: Oxford University Press; 2012.

[29] Van Zyl DH, Joubert DJ. Mandate and negotiorum gestio. In: The law of South Africa. 3rd ed. Vol. 28(1). Johannesburg: LexisNexis; 2020.

[30] Dendy M. Agency and representation. In: The law of South Africa. 3rd ed. Vol. 1. Johannesburg: LexisNexis; 2013.

[31] Harvard Personal Genome Project. Consent form. Harvard PGP [cited 2022 Oct 26]. Available from: https://my.pgp-hms.org/static/PGP_Consent_2021-07-12_online.pdf

